# Niobium on BEA Dealuminated Zeolite for High Selectivity Dehydration Reactions of Ethanol and Xylose into Diethyl Ether and Furfural

**DOI:** 10.3390/nano10071269

**Published:** 2020-06-29

**Authors:** Deborah S. Valadares, Maria Clara H. Clemente, Elon F. de Freitas, Gesley Alex V. Martins, José A. Dias, Sílvia C. L. Dias

**Affiliations:** Laboratory of Catalysis, Chemistry Institute, (IQ-UnB), University of Brasília, Campus Universitário Darcy Ribeiro–Asa Norte, Brasília-DF 70910-900, Brazil; deborahsvaladares@gmail.com (D.S.V.); mrclara01@gmail.com (M.C.H.C.); elonfdf@gmail.com (E.F.d.F.); gesley@unb.br (G.A.V.M.); jdias@unb.br (J.A.D.)

**Keywords:** ^*^BEA zeolite, solid-state dealumination, Nb impregnation, ethanol dehydration, diethyl ether, xylose dehydration, furfural

## Abstract

In this work, we investigated the role of solid-state dealumination by (NH_4_)_2_SiF_6_ (25% Al removal and 13% Si insertion), the impregnation of niobium (10, 18, and 25 wt. %) on dealuminated *BEA (DB) zeolite and their catalytic properties in ethanol and xylose transformations. Among all the studied catalysts, 18%Nb-DB showed increased mesoporosity and external areas. A leveling effect in the number and strength of the proposed two sites (Brønsted and Lewis) present in the catalyst (n_1_ = 0.24 mmol g^−1^, −ΔH_1_ = 49 kJ mol^−1^, and n_2_ = 0.20 mmol g^−1^, –ΔH_2_ = 42 kJ mol^−1^) in the catalyst 18%Nb-DB, might be responsible for its good activity. This catalyst presented the highest selectivity for diethyl ether, DEE (97%) with 61% conversion after 50 ethanol pulses at 230 °C (turnover number, TON DEE = 1.15). These features allowed catalytically fruitful bonding of the ethanol molecules to the neighboring sites on the channels, facilitating bimolecular ether formation through a possible SN_2_ mechanism. The same catalyst was active and selective for transformation of xylose at 180 °C, showing 64% conversion and 51% selectivity for furfural (TON Furfural = 24.7) using water as a green solvent.

## 1. Introduction

Biomass is a sustainable feedstock and its conversion into commercial chemicals, such as ethylene, diethyl ether (DEE), and furfural, represent a good opportunity to protect the environment [1,2]. Heterogeneous catalysis is the key enabler for raw material change, and zeolites play a vital role in shaping the chemical industry. Versatile properties of zeolites, such as well-defined pores and sizes in the molecular dimension, chemical and hydrothermal stability are responsible for important applications [3]. To reach compatibility between the nanoporosity and catalytic selectivity, post-synthesis modifications present success. Typically, post-treatments, such as dealumination [4], impregnation [5], alkali treatment [6], and ion exchange [7], are carried out to increase catalytic performance. The introduction of mesoporosity through a dealumination procedure provide to be a highly efficient diffusional pathway in a confined space [8], as well as to increase the water tolerance at elevated temperatures [9].

In several studies, ethanol can be transformed into ethylene and other short chain hydrocarbons using zeolites [10] and other solid catalysts [11] that present different pore structure and acidity. Gil-Horan et al. [12] studied the effect of incipient impregnation of P, Fe, and Ni in ZSM-5 zeolite for the dehydration of ethanol and subsequent oligomerization of ethylene to short chain olefins. The impregnation substantially improved the activity and stability of the catalysts, and caused significant changes in the strength and concentration of the Brønsted and Lewis acid sites. The P addition allows to obtain a catalyst with acidity predominantly of Brønsted type, and with a higher concentration of total acid sites in comparison with the non-impregnated zeolite, producing some weak Lewis sites. This catalyst was the least selective to olefins, but it produced the highest amount of DEE. In another study, Phung et al. [13] indicated that H-MFI (SiO_2_/Al_2_O_3_ mol ratio = 50) presented >70% yield to DEE at 180–200 °C, with poor ethylene coproduction.

In addition to ethylene, furfural is also considered a building block candidate derived from biomass [14,15,16,17]. The most efficient and inexpensive way to obtain furfural from biomass is by using homogeneous catalysts, such as H_2_SO_4_, HNO_3_, and HCl [2,18]. It is known that these catalysts provide the resinification of furfural, corrosion of industrial installations, reduced yield of the desired product, and generate environmentally hazardous solid and liquid residues [2,18]. This scenario demonstrates the need for research that focuses on selective heterogeneous acid catalysts and improved reaction systems. Some crucial properties of heterogeneous catalysts are the presence of surface acidity, adjusted textural properties and greater hydrothermal stability to produce high selectivity to the desired product. Regarding reaction conditions, the use of solvents, such as water, or mixtures of small amounts of solvents, such as toluene and dimethylsulfoxide (DMSO), and low reaction temperatures (e.g., 100–200 °C), have advantages from an environmental point of view [19]. The catalysts most used in xylose dehydration reactions are zeolites, oxides doped with metals, and nanoparticle mesoporous materials [18,19,20]. The theoretical yield of furfural production from pentosans is 73%, and the current industrial processes operate with ~33% of theoretical yields [19]. Mishra et al. [19] obtained 86 mol% of yield for furfural in xylose dehydration at 150 °C for 12 h, using CuO doped with Zn and water as a reaction medium. Antunes et al. [20] carried out the reaction in a mixture of water/toluene at 170 °C for 2 h using Zr-W-Al-mesoporous nanoparticle catalysts whose furfural yield was 51 mol%.

According to the above, the aim of the present work was to investigate the niobium impregnation on dealuminated *BEA zeolite and its influence over the conversion of ethanol to diethyl ether and the conversion of xylose to furfural. To unravel the effects on the dealumination procedure and Nb impregnation on both reactions, we used infrared (Fourier transform infrared spectroscopy (FTIR)), X-ray diffraction (XRD), and solid state ^27^Al and ^29^Si nuclear magnetic resonance (MAS NMR) to look for changes in the structural characteristics; N_2_ sorption to follow the textural properties; pyridine (Py) adsorption to identify the Brønsted and Lewis acid sites, and liquid phase Py adsorption microcalorimetry to quantify this information.

## 2. Materials and Methods

### 2.1. *BEA Dealumination and Niobium Aqueous Impregnation

*BEA zeolite was obtained from Zeolyst International (CP814E) in the ammonium form with a SiO_2_/Al_2_O_3_ mole ratio = 25. This zeolite was subjected to solid state dealumination, using ammonium hexafluorosilicate, (NH_4_)_2_[SiF_6_], (AHFS, Aldrich, St. Louis, MO, USA, 98%), in a sufficient amount to remove theoretically 70 mol% of the total aluminum. Both solids were mixed for 10 min using a mortar and pestle, and placed in a closed desiccator containing a saturated solution of ammonium chloride (Sigma-Aldrich, St. Louis, MO, USA, >99.5%), and under atmospheric pressure (24 h). Subsequently, the mixture was taken to a muffle furnace (3 h) at 190 °C. After that, the material was washed with 50 mL of an ammonium acetate solution (five times), and once with 100 mL of deionized water at room temperature. During this procedure, the ammonium acetate solution (0.5 mol L^−1^) and water were evaluated, after passing through the material, for the pH measurement and the formation or absence of precipitate in contact with the NaOH solution (1 mol L^−1^). The material was dried at 120 °C (14 h), ground, and calcined at 550 °C (8 h). To obtain protonic zeolite HB, the NH_4_BEA zeolite was calcined at 550 °C (8 h) [9]. In a previous work of our group [9], Energy Dispersive X-ray fluorescence (EDXRF) results indicated that the Si/Al ratio (molSi molAl^−1^) increased from 12.5 (NH_4_BEA) to 18.7 after dealumination procedure described above. The results showed that 25% of Al was removed from parent zeolite, while 13% of Si was added to the sample from the dealuminating agent (AHFS).

For the impregnation of niobium in *BEA zeolite, an aqueous solution of ammonium niobium oxalate from Companhia Brasileira de Mineração e Metalurgia (CBMM, Araxá, MG, Brazil) was added to the *BEA dealuminated in the required quantity to produce 10, 18, and 25 wt. % of Nb_2_O_5_ supported on the zeolite. Then, it was kept at 80 °C, until the complete evaporation of the water. The remaining solid was ground and calcined at 550 °C (8 h) [9]. Table 1 shows the nomenclature of the catalysts.

### 2.2. Characterization

A Thermo Scientific spectrometer (Nicolet, model 6700, Madison, WI, USA) was used to obtain the Fourier transform infrared spectroscopy (FTIR) spectra. FTIR pellets were prepared with 1 wt. % zeolite sample diluted in KBr (Merck, Darmstadt, Germany, >99%) under ambient conditions. The spectral resolution was 4 cm^−1^, and 256 scans were performed.

X-ray diffraction patterns (XRD) were obtained in a Bruker powder diffractometer (model D8 Focus, θ-2θ, Germany), radiation from a copper tube (Kα = 1.5406 Å, 40 kV, 30 mA), and a scanning rate of 2° min^−1^ (2θ from 5 to 50°, step of 0.02°).

The textural information was obtained by adsorption-desorption isotherms [4,21], where each sample contained in an evacuated sample tube was cooled to cryogenic temperature (–196 °C), and was then exposed to N_2_ gas (White Martins, 99.999%, Brazil) using a surface area and porosimetry analyzer (Micromeritics, ASAP model 2020C, Atlanta, GA, USA). The samples were degassed, before the analysis with heating at 300 °C (4 h) and evacuation (target pressure of 10 μm Hg). The BET equation (range of partial pressure (P/P_o_) from 0 to 0.1), t-plot method, and BJH method were used to describe the experimental isotherms, and to calculate the catalyst crystallinity [9]. We considered that the *BEA zeolite, in its protonic form, was the standard sample, and had 100% crystallinity and the impregnation percentages of the supported Nb were discounted.

Solid-state ^27^Al and ^29^Si magic angle spinning nuclear magnetic resonance (MAS NMR) spectra were obtained in a Bruker Avance III HD Ascend 600 MHz spectrometer, Germany (14.1 T, 10 kHz spin rate). A CP/MAS probe (4 mm) was used for solids, and the catalysts were packed into a 4 mm hollow cylindrical zirconia rotor, with the following conditions for each nucleus: (i) ^27^Al MAS NMR: 156.4 MHz, 0.4 μs pulse duration, 1 s pulse interval, 2000 acquisitions for each spectrum), and the external reference was solid, Al(H_2_O)_6_Cl_3_ (Sigma-Aldrich, St. Louis, MO, USA, 99%) (δ, 0 ppm); (ii) ^29^Si MAS NMR: 119.3 MHz, 4.25 μs pulse duration, 20 s pulse interval, 512 acquisitions for each spectrum, and the external reference was Si(CH_3_)_4_ tetramethylsilane (Sigma-Aldrich, St. Louis, MO, USA, >99.9%) (δ, 0 ppm).

We used gas phase pyridine (Sigma-Aldrich, St. Louis, MO, USA, sure seal, 99.8%) adsorption, followed by FTIR analysis, to identify the nature of acidity of the catalysts [9,22]. The FTIR spectra were obtained using 10 wt. % catalysts diluted in KBr.

Microcalorimetric titrations were performed in a modified (magnetic stirring) isoperibol calorimeter (ISC, Model 4300, Calorimetry Sciences Corporation, Linden, UT, USA). The equipment was coupled to a computer, and the experiments registered according to the provided software. Each catalyst was dried at 300 °C for 2 h in a glass reactor under vacuum before measurements. Then, it was moved to a glove box under N_2_ (99.99%, White Martins/Praxair, Brazil), weighted (0.5 g), and transferred to the calorimetric cell (25 mL), followed by the addition of 25 mL of anhydrous cyclohexane (Vetec, Brazil, >99%, fresh distilled over P_2_O_5_). In a glove bag filled with N_2_, a calibrated gas tight syringe (Hamilton, NV, USA, 5 mL) was filled with standardized pyridine solution in cyclohexane (0.1002 mol L^−1^). Next, both components (cell and syringe) were inserted into the calorimeter holder, which was immersed in a thermal bath regulated at 25 °C. Before any experiment, it was allowed the system to equilibrate (1 h). The addition of the pyridine solution was done incrementally (e.g., 0.1 mL), using the injection system of the calorimeter. After each injection of pyridine, the heat evolved from its reaction with the solid in suspension was measured, considering the initial and final baseline observed in the software. The interval between additions was 4 min, which was sufficient for the system to equilibrate. To calculate the heat for each injection, the equivalent energy of the system was obtained using a calibration curve, performed before and after each titration. The heat of diluted pyridine added to cyclohexane was measured separately and considered negligible. Each titration was repeated two times for each catalyst, in agreement with previous work [22].

The calorimetric data (heat evolved versus py added) were used to calculate the number of sites (n_i_) and the enthalpies (ΔH_i_) of the catalysts. A model with two sites (n_1_ and n_2_) was chosen to represent the acid sites on these catalysts, based on analogous systems studied by our group [23]. The curve of heat evolved versus Py added was taken in two linear portions, which were fitted by linear regression. The enthalpies (ΔH_1_ and ΔH_2_) were obtained from the slope of each regression. The number of sites (n_1_ and n_2_) were calculated from selected enthalpy ranges. Two lines are proposed and split dividing the points. The decision of where one curve ends (n_1_) and another begins (n_2_) is made by linear regression of the points. When adding a point to the curve, if it is observed a deviation from linearity (R^2^ moves away from 1), then that point is considered to belong to another curve (the other acidic site, n_2_).

### 2.3. Ethanol Catalytic Dehydration

The ethanol catalytic dehydration reaction was tested, from pulse 1 to 50, in a pulsed-flow fixed-bed microreactor coupled to a gas chromatograph system and with a flame ionization detector (Shimadzu GC-FID, Kyoto, JP, model 2010; column Shimadzu CBP1 PONA-M50-042, with dimensions of 50 m × 0.15 m × 0.33 μm). In each analysis, 0.5 μL of ethanol (Vetec, Brazil, > 99.8%) was injected into the reactor (liner) containing 10 mg of the catalyst. The following experimental conditions were used: pressure of 95.6 kPa, total flow of 6 mL min^−1^, linear velocity of 6.4 cm s^−1^, purge flow of 1 mL min^−1^, split ratio of 49, helium as carrier gas, and the flame (FID) temperature was 250 °C. The catalysts were treated in situ at 250 °C (30 min), and the reaction analyses were performed at 230, 250, and 300 °C. The detailed analysis of the products can be found elsewhere [1].

After the catalytic dehydration reactions, elemental analyses were conducted on the samples in a CHN system (PerkinElmer, series II, model 2400, MA, USA). We deposited 2.5 mg of the material in tin folding crucibles using an electronic ultra-microbalance (PerkinElmer AD-6 Autobalance, with sensitivity of 0.1 μg). These analyses were conducted to quantify the amount of carbon residues formed after the dehydration reaction [9].

Conversion of ethanol and selectivity for DEE were calculated by Equations (1) and (2), respectively, where n is the number of moles.

(1) $$\mathrm{Conversion}\;(\%)=\frac{{n_{\mathrm{ethanol}}}_{\mathrm{initial}}-{n_{\mathrm{ethanol}}}_{\mathrm{final}}}{{n_{\mathrm{ethanol}}}_{\mathrm{initial}}}\;\times \;100$$

(2) $$\mathrm{Selectivity}\;(\%)=\frac{n_{\mathrm{DEE}}}{{n_{\mathrm{ethanol}}}_{\mathrm{initial}}-{{\;n}_{\mathrm{ethanol}}}_{\mathrm{final}}}\;\times \;100$$

The turnover number (TON) for ethanol conversion (TON_CONV._) and DEE selectivity (TON_DEE_) was calculated following Equations (3) and (4) below, respectively, where n is the number of moles.

(3) $${\mathrm{TON}}_{\mathrm{CONV}}=\frac{n_{\mathrm{converted}\;\mathrm{ethanol}}}{n_{\mathrm{acid}\;\mathrm{sites}}}$$

(4) $${\mathrm{TON}}_{\mathrm{DEE}}=\frac{n_{\mathrm{DEE}}}{n_{\mathrm{acid}\;\mathrm{sites}}}$$

### 2.4. Catalytic Conversion of Xylose

Each reaction was carried out in Teflon autoclaves with a stainless steel outer jacket under magnetic stirring and a sand bath. The proportion of catalyst:xylose used in the reaction was 1:5, and the reaction took place in an aqueous phase (12.44 mL of deionized water). The mass of D-xylose (Sigma-Aldrich, St. Louis, MO, USA, >99%) was 0.31 g, and the mass of the catalyst was 0.0621 g. The reaction temperatures were 160 °C and 180 °C and the TOS (time on stream) 2 h.

The reactor was cooled, and the separation of reaction products from the catalyst was completed by centrifugation at 4000 rpm. The supernatant was filtered through a syringe filter with a 25 mm CHROMAFIL^®^ PA polyamide membrane and a pore size of 0.20 µm. The samples were placed in 2 mL vials and kept under refrigeration (5 °C).

### 2.5. Analysis of Catalytic Conversion of Xylose to Furfural by HPLC-RID/PDA

The products were analyzed on the same day, or one day after the reactions, to avoid the degradation of the product. The products were analyzed by high performance liquid chromatography (HPLC, LC-20A Prominence, Shimadzu, Kyoto, Japan) with detection by refractive index (RID) and a photodiode matrix (PDA) using tungsten and deuterium lamps as the source. A solution of H_2_SO_4_ 25 mmol L^−1^ was used as a mobile phase in the proportion of 50% mobile phase to 50% deionized water (v/v). For the separation of the products, a chromatographic column of ionic exclusion Shim-pack SCR-102 H was used, whose stationary phase consisted of a sulfonated styrene polymer of type H. The dimensions of the column included an internal diameter of 8 mm, length of 30 cm, and particle size of 7 µm. The analyses were performed under a flow of the mobile phase of 1 mL min^−1^ and injection volume of the samples of 1 µL. Each run lasted 25 min with the column temperature (oven) at 80 °C.

**Figure 1:**
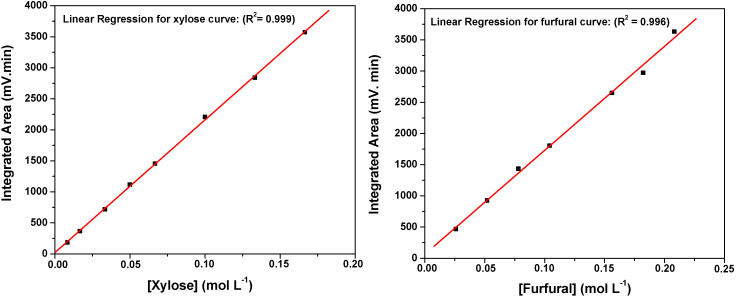
Analytical curves of xylose and furfural obtained by high performance liquid chromatography (HPLC)-refractive index (RID).

The linearity in HPLC detection for both the reactant and main product molecule was verified (Figure 1). The standards were analyzed by HPLC-RID. The analytical curves were constructed using integrated area of the peak of standard chromatograms (xylose and furfural, Figure S1). Through the analytical curve of xylose, it was possible to determine its conversion. Meanwhile, the production of furfural w/v (%) was obtained through the analytical curve of furfural (Sigma-Aldrich, St. Louis, MO, USA, 99%) and its straight-line equation.

Conversion and selectivity to furfural were calculated by Equations (5) and (6), respectively, where n is the number of moles.

(5) $$\mathrm{Conversion}\;(\%)=\frac{{n_{\mathrm{xylose}}}_{\mathrm{initial}}-{n_{\mathrm{xylose}}}_{\mathrm{final}}}{{n_{\mathrm{xylose}}}_{\mathrm{initial}}}\;\times \;100$$

(6) $$\mathrm{Selectivity}\;(\%)=\frac{n_{\mathrm{furfural}}}{{n_{\mathrm{xylose}}}_{\mathrm{initial}}-{n_{\mathrm{xylose}}}_{\mathrm{final}}}\;\times \;100$$

The turnover number (TON) for xylose conversion (TON_CONV.)_ and furfural selectivity (TON_Furfural_) was calculated following Equations (7) and (8) below, respectively, where n is the number of moles.

(7) $${\mathrm{TON}}_{\mathrm{CONV}.}=\frac{n_{\mathrm{converted}\;\mathrm{xylose}}}{n_{\mathrm{acid}\;\mathrm{sites}}}$$

(8) $${{\mathrm{TON}}_{\mathrm{DEE}}}_{\;}=\frac{n_{\mathrm{Furfural}}}{n_{\mathrm{acid}\;\mathrm{sites}}}$$

## 3. Results and Discussion

In the present work, three percentages of the Nb_2_O_5_ (10, 18 and 25 wt. %) were impregnated on the *BEA zeolite dealuminated (Si/Al = 18.7).

### 3.1. Characterizations

**Figure 2:**
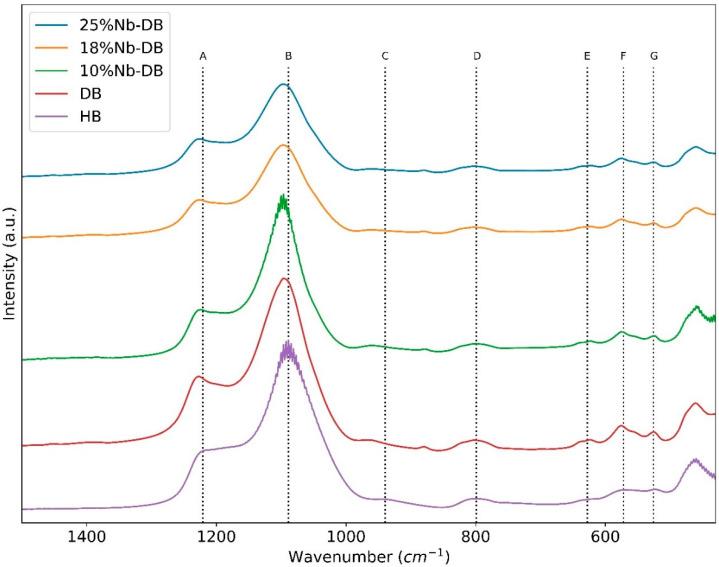
Fourier transform infrared spectroscopy (FTIR) spectra of the catalysts, indicating the main bands A to G.

Figure 2 shows the FTIR spectra and Table 2 indicates the main absorption bands identified for each catalyst, according to the literature [24,25].

The asymmetric vibration on the SiO_4_, identified at approximately 1220 cm^−1^ (column A), and the asymmetric vibration between the zeolite tetrahedral and the oxygen atoms (TO, column B) were shifted to higher wavenumbers after the dealumination (increase of Si/Al ratio), in agreement with the literature [26,27]. However, no significant change was observed after the impregnation of Nb, within the experimental FTIR resolution (4 cm^−1^).

In addition, the vibration corresponding to the Si–O bound (column C) shifted to higher wavenumbers for dealuminated sample (DB), which is also sensitive to Si/Al ratio [27]. For the samples with Nb, if it had been incorporated into the zeolitic network, the wavenumber found would have been higher (960–970 cm^−1^) [28], but no significative shift was observed. Thus, we inferred that most of the impregnation of Nb occurred in the form of Nb_2_O_5_, outside the framework in all catalysts. The characteristic wavenumber of the symmetric vibration of the Si–O–Si connections of the zeolitic network (column D), and the vibrations that occurred in the six member zeolite rings (columns E, F and G) remained in the expected range, providing an indication that the dealumination did not compromise the zeolite structure [24,25,26,29].

**Figure 3:**
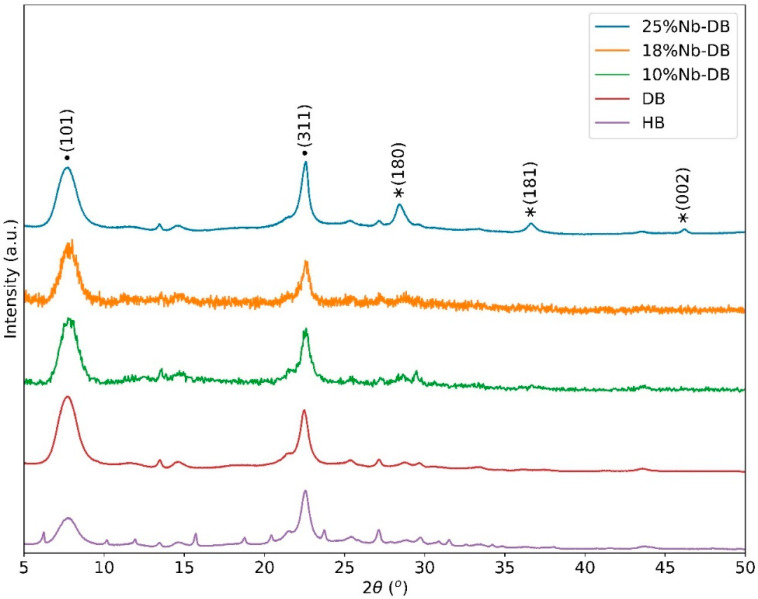
X-ray diffraction patterns of the catalysts (• refers to *BEA and * to T-Nb_2_O_5_).

The results of the XRD (Figure 3) showed that the peaks at 2θ = 7.9° and 22.5° corresponded, respectively, to the planes (101) and (311), characteristic of the polymorph A of zeolite *BEA. The presence of these peaks in all catalysts is an indication that there were no significant changes with respect to the structure of the support, even after performing the dealumination procedure and niobium aqueous impregnation. Furthermore, as it was not possible to identify the presence of characteristic niobium oxide signals, it is also possible to infer that there might be a good dispersion of it on the support, or that there was the formation of amorphous species small enough that they could be not be detected by powder XRD [23,30]. We observed a decrease in the crystallinity content of the catalysts from 100% for HB, 87% for DB, 85% for 10%Nb-DB, 77% for 18%Nb-DB, and 85% for 25%Nb-DB, which did not significantly compromise the zeolite structure, as discussed previously.

Only the 25%Nb-DB catalyst showed the presence of three signals with relative low intensity at 2θ = 28.4°, 36.6°, and 46.2°, corresponding to the planes (180), (181), and (002) of the crystallographic T-phase of Nb_2_O_5_ (orthorhombic), respectively, which can be addressed to the incorporation of metal oxide outside the zeolitic framework [24,31,32]. The difference in the relative peak intensities of pure T-Nb_2_O_5_ to 25%Nb-DB is related to the higher exposition of plane (180) on the supported one.

Table 3 presents the results of the textural analysis obtained from the isotherms for all catalysts (Figure S2). Analyzing the obtained textural data (Table 3), the specific surface area (S_BET_) decreased after dealumination and Nb impregnation, which may indicate the obstruction of the pores by the presence of extra framework aluminum species (that cannot been completely removed after washing) and the presence of the supported material. This same trend was observed in the external surface area (S_Ext_), except for the catalyst 18%Nb-DB, which showed a slight increase. The microporous area (S_Micro_), and the volume of micropores (V_μ_), decreased with the modifications exerted on the catalysts, but there was an increase in the diameter of the mesopores (D_Meso_), due to dealumination, which can influence the access of the reactants to the active sites.

**Figure 4:**
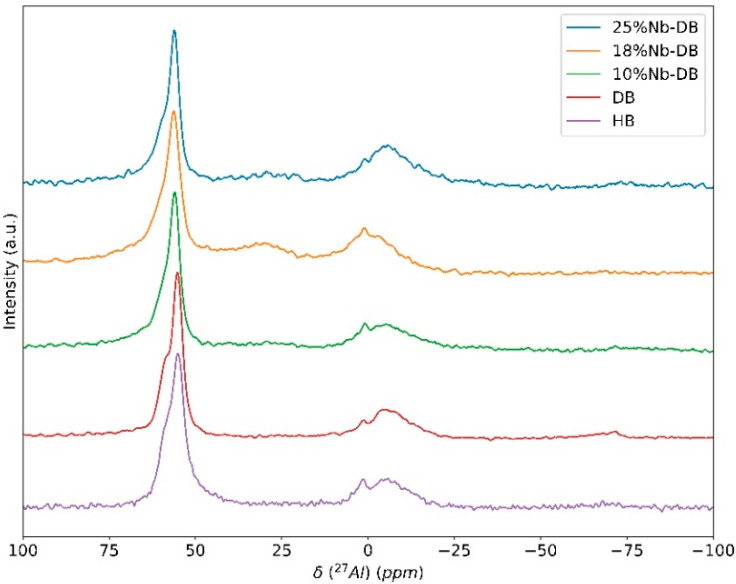
^27^Al MAS NMR spectra of the catalysts.

The signals obtained with the ^27^Al MAS NMR (Figure 4) were integrated into two distinct ranges of chemical shifts: 40 to 80 ppm (tetrahedral Al, Al Td) and −22 to 22 ppm (octahedral Al, Al Oh). The shoulders that appear in the main signals at 60 ppm and 0 ppm have been assigned as tetrahedral Al in the polymorphous A and B of zeolite *BEA [33]. Table 4 (columns 1 and 2) shows the relative distribution of Al species in the catalysts. The HB catalyst had approximately 64% Al Td in its structure. After dealumination, there was an increase in the amount of Al Td, and a decrease in the Al Oh, which was possibly a consequence of the rearrangement of Al Oh into Al Td. With the increase in the percentage of Nb over DB, there was an increase in the percentage of Al Oh. In catalytic reactions, Al Td can be characterized as a strong Brønsted acid site and Al Oh as a Lewis acid site (located outside the framework) [4,34]. Thus, the addition of niobium on DB zeolite promoted a decrease of Al atoms from the framework during thermal treatments, as observed by supported Nb_2_O_5_/ZSM-5 [35,36]. As a result of this, we inferred that dealumination decreased the relative amount of strong Brønsted acid sites, and increased the number of Lewis acid sites. The aqueous impregnation with Nb enriched the surface of the catalyst with an overlayer of Nb_2_O_5_, which can also contribute to the Lewis acid sites.

**Figure 5:**
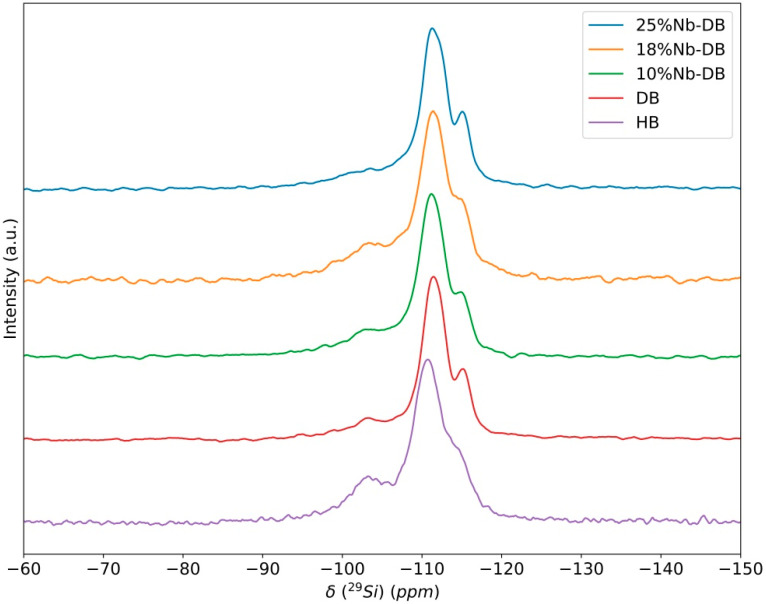
^29^Si magic angle spinning nuclear magnetic resonance (MAS NMR) spectra of the catalysts.

Figure 5 shows the experimental ^29^Si MAS NMR spectra. The deconvoluted signal spectra are displayed in Figure S3. The relative distribution of the main signals is in Table 4. After the modifications made to the catalysts, there was no significant shift in the Q^3^ signals around −102 ppm, which refers to Si (1Al, 3Si or SiOH) in different environments and neither in Q^4^ (around −111 and −115 ppm, which can be associated with the Si (0Al) environment. Zeolite *BEA presented nine different crystallographic sites [37], which can cause different chemical shifts to the same Si (nAl) environment [28,34,38]. The major modification of Q^n^ distribution was from HB to DB, with the decreased amount of Q^3^. A possible contribution in Q^4^ that should not be totally ruled out is the deposit of silica on the surface of the DB crystallite. This silica would be formed by the hydrolysis of SiF_4_ that results from the decomposition of (NH_4_)_2_SiF_6_ in the process of dealumination [39]. After addition of Nb, the Q^n^ distribution was about the same of HB, which indicated that no strong modification of the zeolite structure was detected, as confirmed by FTIR and XRD measurements.

**Figure 6:**
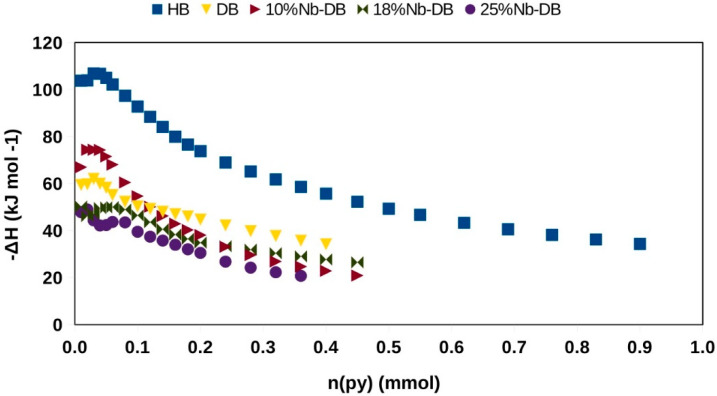
Calculated enthalpy difference (ΔH) versus the number of mmoles of Py added.

The gas phase pyridine adsorption results identified FTIR bands related to the Brønsted (1545 cm^−1^) and Lewis (1447 cm^−1^) sites (Figure S4). The calorimetric titration provides the heat released after adding the probe molecule (Py) to the catalyst, which is related to the interaction between the base and the acidic sites (Brønsted and Lewis) present on the surface. The curves corresponding to the calculated enthalpy difference (ΔH) versus the number of mmoles of Py added was plotted in Figure 6. The curves indicated that pyridine initially reacted preferentially with the stronger Brønsted acid sites (site 1), due to the most negative free energy associated with these sites [40]. In the middle of the titration, there was a simultaneous interaction of the base with either Brønsted sites or Lewis sites. At the end of the titration, the probe molecule interacted mainly with Lewis sites and weaker hydrogen bonding sites, which may contribute to lower the average strength of site 2 [40]. It can be observed that HB has stronger acid sites than the other catalysts, and many more sites were accessible to pyridine. Quantitatively, the strength of the sites may be divided in approximately two ranges: i) the highest enthalpies (ΔH_1_) for all catalysts, which extends up to 0.15 mmoles; ii) the lower enthalpies (ΔH_2_), which is from 0.15 to 0.45 mmoles of the Py added. For the HB zeolite, the range is extended up to approximately 0.70 mmoles. Clearly, it can be considered an intermediate range of enthalpies, where there is a simultaneous interaction of pyridine with either the Brønsted or Lewis sites. Thus, based on the proposed method of calculation, the different number of acid sites (n_1_ and n_2_) and their respective enthalpies (ΔH_1_ and ΔH_2_) were obtained (Table 5).

It could be noted that the 18% Nb-DB catalyst showed a similar number (mmoles) of the two acid sites with average enthalpy—(ΔH_1_ + ΔH_2_) of ~−45 kJ mol^−1^) and more external area among the supported catalysts.

### 3.2. Ethanol Catalytic Dehydration

Before the dehydration reactions, three runs with ethanol (without catalyst) were carried out at the three reaction temperatures used. After that, we calculated an average of the amount of ethanol that, in fact, reached the catalyst and this amount was adopted as the total ethanol that went to the catalytic bed. From the obtained results, we carried out selectivity calculations for diethyl ether (DEE), ethylene, and other products that may have been formed. The average error in this procedure was 3%, which can be taken for the conversion and selectivity results. The percentage of the conversion of ethanol in pulses 1 and 50, at the different temperatures, are shown in Figure S5, whereas the conversion and turnover numbers are presented in Table 6. In a former study [9], we confirmed that among the used catalysts, HB was the most active to the conversion of ethanol into ethylene, with percentages higher than 90%. Even at pulse 50, where it was expected to have a greater amount of coke in the catalyst, which consequently could reduce the catalytic performance, the conversions remained above 90%. The dealumination decreased the catalytic performance for DB since it presented conversions between 69% and 81%. Higher temperatures favored the conversion, and thus the reaction at 300 °C showed about 12% higher conversion than the reaction at 230 °C. Based on TON, DB was more active at 300 °C (1.7 compared to 1.5 from HB) and, generally, it was noted that TON increased with the temperature, since the conversion of ethanol also increased, and the total number of acid sites decreased (0.40 for DB compared to 0.61 for HB).

With these results, it is acceptable to infer that the Brønsted and Lewis acid sites directly influenced the catalytic dehydration. As the HB catalyst had not undergone any modification, it was the one with the most Brønsted acid sites (−ΔH_1_ = 105 kJ mol^−1^ related to the Al Td species and bridge silanol groups). Likewise, higher temperatures also contributed to the catalytic performance. The dealuminated zeolite presented fewer strong acid sites (n_1_ = 0.16 mmol g^−1^ compared to 0.21 from HB), and showed a lower catalytic conversion; however, this performance increased in the reactions at higher temperatures. The niobium addition on the support (Nb-DB) showed generally higher conversion with Nb loading, which was possibly due to the gain of the Lewis sites (−ΔH_2_ average values around 45 kJ mol^−1^). The 18%Nb-DB catalyst showed an increase in the catalytic performance (more Lewis acid sites) relative to 10%Nb-DB. Finally, the catalyst with the largest amount of supported Nb had low catalytic activity that was only compensated at 300 °C.

Much more significant than the conversion was the selectivity. In this sense, calculated TON for DEE (Table 6 and Figure S6) illustrates, respectively, the formation of DEE and ethylene in the first and fiftieth injection of ethanol through the catalytic bed. No other products were detected. Considering pulse 1, the selectivity for ethylene was greater than 96% with the HBEA catalyst, reached 100% when the dehydration was carried out at 300 °C, and did not produce any traces of ethylene oligomers, as observed before [9]. The selectivity of the DB catalyst was greater than 64% at 230 °C for DEE, but declined with increasing temperature. The increase in selectivity for DEE was verified with the impregnation of niobium oxide and at lower temperature reactions, despite presenting 17% selectivity at 300 °C. The highest selectivity (95%) for DEE was obtained with the 18%Nb-DB catalyst in the reaction at 230 °C, also confirmed by TON calculation. The catalyst with the highest loading of Nb (25%Nb-DB) showed 86% selectivity at 230 °C, and only 39% at 300 °C.

From these results, it is possible to infer that the DEE production was influenced by diffusion of the reagent through the catalyst. Dealumination increased the amount of mesopores (which favors the access of reactants to the acidic sites in a bimolecular reaction), increased the hydrophobicity of the catalyst (eliminating possible interactions of water with acidic sites) [41], and decreased the amount of Brønsted acid sites (which favor the ethylene formation)—factors that directly influence the selectivity for DEE. In addition, the low WHSV (57 h^−1^) during the reaction favored the access of ethanol to the Lewis acid sites (derived from the presence of supported oxide and extra framework aluminum species). The results of these combinations made it possible to produce DEE in a highly selective manner. The percentage of carbon (coke) formed and deposited on the catalysts after the dehydration reaction (50 pulses) ranged from 4.1% for HB (230) to 0.62% for 18%Nb-DB (230). The lower coke formation of 18%Nb-DB is probably because of lower number of Brønsted acidic sites than HB, which are known to facilitate the formation of high content of carbon deposits [1,9,13].

A brief comparison with other results in the literature is provided. Yang et al. [11] used catalysts of metal organic framework with nodes of Zr_6_O_8_ to evaluate the dehydration of ethanol at 200 and 250 °C. Under a conversion regime lower than 10% (differential reactor), they obtained 100% selectivity for DEE, but the conversion quickly declined after 50 min. Gil-Horán et al. [12] working at 350 °C in a isothermal fixed-bed reactor, obtained about 18% of DEE after 5 h reaction. They used modified ZSM5 (Si/Al = 80) with P and Ni as catalysts. Phung et al. [13] studied several protonic zeolites at reaction temperatures of 180 to 200 °C. They found that H-MFI (Si/Al = 50), at a low conversion regime, had more than 70% selectivity for DEE, and only 1.4% ethylene yield. Thus, these few examples show that our results are promising for further developments.

### 3.3. Catalytic Conversion of Xylose

**Figure 7:**
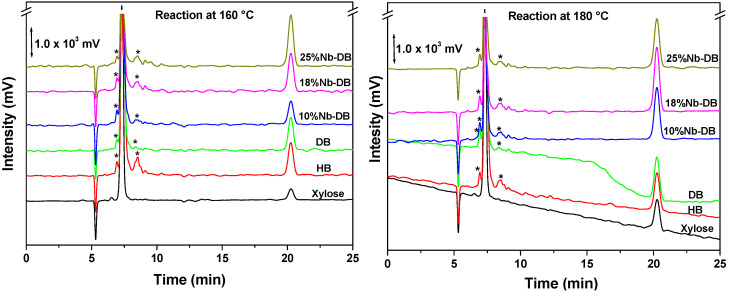
HPLC-RID chromatograms generated in the catalytic reactions (* refers to by-products).

The chromatograms obtained by HPLC-RID for all catalysts applied in the reaction of xylose to furfural at temperatures of 160 and 180 °C are shown in Figure 7. The retention time of the xylose peak was around 7.5 min, furfural was at 20.1 min and by-products peaks at 6.9 min (immediately before the xylose peak and between xylose and furfural peaks).

**Figure 8:**
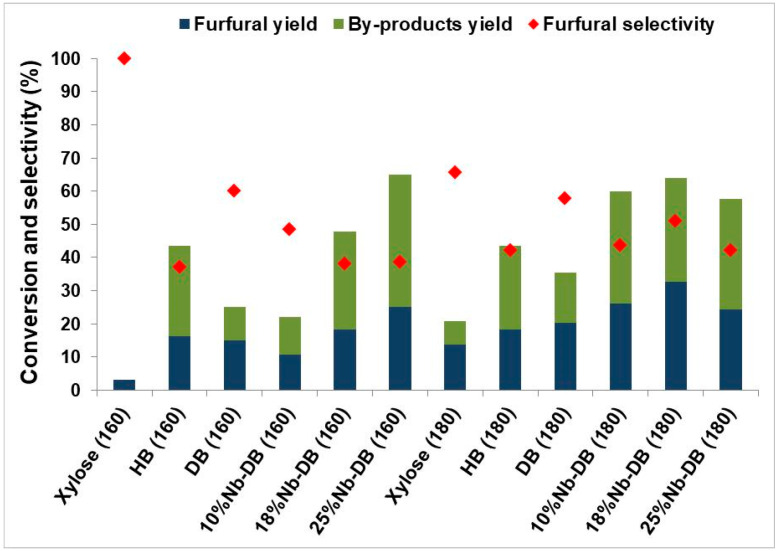
Conversion (%) of xylose and selectivity (%) for furfural.

In the reaction without catalyst (Figure 7), there was low conversion (intense peak due the presence of the xylose), but high selectivity for furfural, which can be considered as 100%, due to the absence/insignificance of by-product peaks in the xylose chromatogram (160 and 180 °C). The conversion improved by also increasing the temperature to the catalytic reactions, and the selectivity remained almost the same. This information corroborates that of Figure 8, which shows that the temperature of 160 °C is still low to obtain good results from the conversion of xylose. At this temperature, despite the low conversion of xylose (~25%) and furfural production (~15%), the DB catalyst showed the highest selectivity for the desired product (~60%). The 25%Nb-DB catalyst also showed excellent results. Although the selectivity (~40%) was lower than the DB catalyst, the conversion was approximately 65%, and with a furfural production of 25%. The increase in temperature mainly influenced the conversion results of xylose.

The reaction temperature was important to the considerable change in the results of conversion and selectivity for the analysis of the reaction product without a catalyst. At 160 °C, there was no significant conversion, while at 180 °C, a higher selectivity for furfural was observed (~66%), although the conversion was only 21% and the production of furfural was 13%. Among all the catalysts applied in the reaction, the 18%Nb-DB (180 °C) catalyst was the one that showed a conversion of 64% of xylose and the highest yield of furfural (~35%).

**Figure 9:**
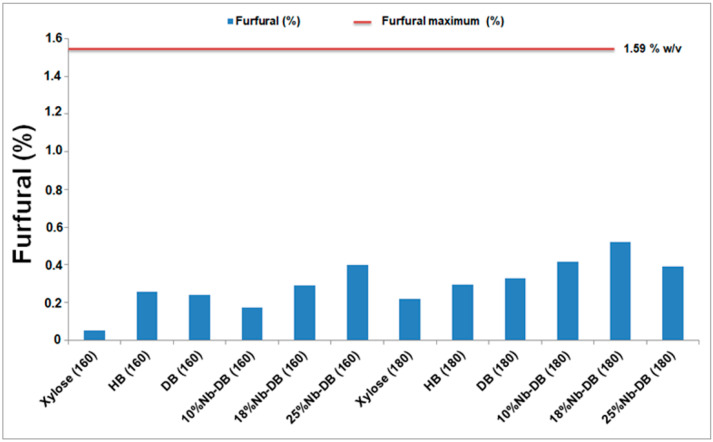
Produced furfural (%) during the reactions in the reagent solutions at different temperatures and using different catalysts. Maximum (%) of furfural (**—**) that could be produced in the reagent solutions, under our experimental conditions.

The production of furfural w/v (%) can be analyzed for each catalyst in Figure 9. The maximum possible percentage of furfural production in the reactant solution, considering the initial mass of the xylose (0.31 g), was around 1.59 w/v (%). According to Figure 9, the furfural production increased with temperature and the highest production was for the 18%Nb-DB (180 °C) catalyst with ~0.5 w/v (%). This catalyst also had an increased number of Lewis acid sites.

Studies show that Brønsted acid catalysts, such as HB, convert xylose directly to furfural [42,43,44]. Therefore, good results are expected for conversion and selectivity using this catalyst. In fact, there was a high conversion of xylose (~44%) from HB at 160 and 180 °C (Figure 8). These conditions extended to selectivity (~37% and 42%, respectively) if compared to the reaction without catalyst (xylose), which only showed a considerable conversion in the reaction at 180 °C. Due to the similarity in the HB results at both temperatures, we concluded that this parameter was not the determining factor, but the Brønsted acid sites were. Despite demonstrating good conversion and selectivity results by directly transforming xylose, the high acidity of Brønsted will not only form furfural as a product, but also the selectivity of the reaction will be impaired by this factor, as can be seen in Figure 7 and Figure 8. The reactions carried out with HB catalyst at both temperatures (Figure 7) indicate significant peaks (asterisks) of possible by-products.

**Scheme 1:**
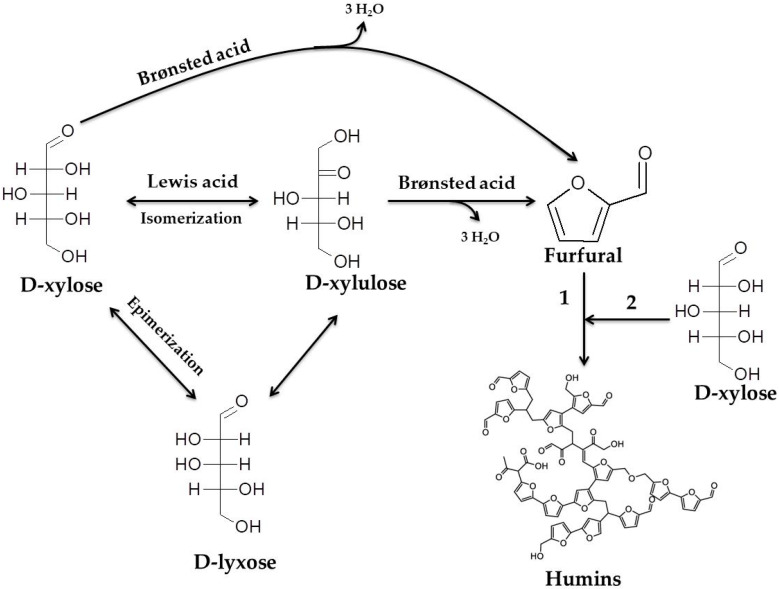
Xylose dehydration reaction and humins formation reactions based on [42,43,47].

The presence of the Lewis acid sites, predominant in the impregnated Nb catalysts, altered the path in the conversion of xylose to furfural in relation to the reaction with stronger Brønsted acid sites (Scheme 1). Before the conversion of xylose to furfural, its isomerization to xylulose or epimerization to lyxose occurs [45]. The dehydration of xylulose increases furfural yield when compared to xylose dehydration under similar reaction conditions [43]. The combination of the Brønsted and Lewis acidic sites with moderate strength is essential to form enediol intermediates resulting in better conversion and selectivity [43,44,45,46]. The reaction temperature, although it did not influence the catalyst with a high number of Brønsted acid sites (HB), caused a considerable increase in the conversion for those catalysts with greater Lewis acidity. It is possible to notice in Figure 8 that the conversion of xylose via DB catalyst increased from 25% to 35%; for 10% Nb-DB from 22% to 60% and for 18% Nb-DB from 48% to 64%, with increasing reaction temperature. Generally, the increase in the proportion of Nb_2_O_5_ impregnated on the catalysts increased the conversion of xylose at 160 and 180 °C. Some by-products can be the intermediates (xylulose and lixose mentioned above), due to the proximity in the retention time in relation to the peak of xylose obtained by HPLC-RID. In addition to these by-products, humins can also be generated by the reaction of furfural with xylose (Reaction 2, Scheme 1) or by reacting with itself (Reaction 1, Scheme 1), but it is not possible to determine them by HPLC [42,47].

Table 7 shows the calculated turnover number (TON) based on conversion of xylose and the formation of furfural, and the respective acidity of the catalysts (total number of acid sites, n_1_ + n_2_). It can be observed that TON increased with temperature and also with Nb loading. Moreover, the 18%Nb-DB catalyst showed the highest TON for yielding furfural at 180 °C. It is important to remember that these reactions were performed in water.

**Figure 10:**
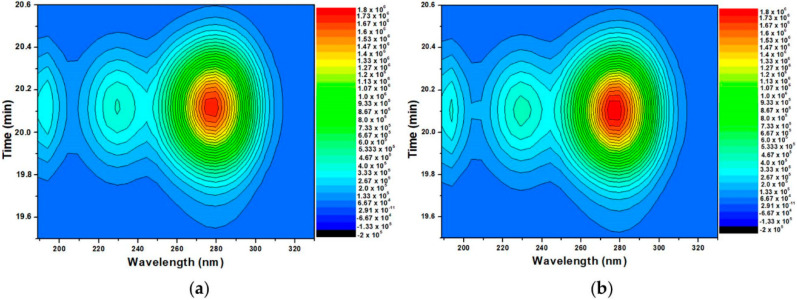
(**a**) Contour curves of the furfural pattern 0.5 (w/v%); (**b**) The analysis of the product obtained after the reaction with the catalyst 18%Nb-DB (180 °C). The *X*-axis represents the UV-Vis absorbance spectrum, and the *Y*-axis represents the retention time in the chromatogram.

Figure 10 shows a comparison between the contour graphics of the furfural pattern (0.5 w/v%) and most of the products obtained through the xylose conversion reaction at 180 °C, using the 18%Nb-DB catalyst. These curves were constructed with the 3D data from the analysis by HPLC-PDA. In both graphs (a and b), the furfural retention time was around 20.1 min. In addition, the wavelength that presented the highest absorbance intensity for furfural (red region in the curves) was around 275 nm. This wavelength is commonly used to quantify furfural [48,49]. The information in the graph allows us to conclude that the product obtained from the reaction was actually furfural, and its concentration for the reaction at 180 °C was around 0.5 (w/v%), confirming the data in Figure 9.

Xylose conversion reactions using solvents, such as tetrahydrofuran (THF), dimethyl sulfoxide (DMSO), toluene, butanol, and γ-valerolactone (GVL), provide higher conversion and selectivity values [45,46,50,51,52]. Therefore, reactions carried out in a medium where only water is used as the solvent is a challenge. This type of reaction is desired from the point of view of green chemistry, as well as the need to avoid homogeneous acid catalysts (e.g., H_2_SO_4_, HCl). Table 8 lists some catalysts and reaction conditions used in the literature, including two of the best results obtained in this work.

Comparing the conversion results of xylose and the selectivity to furfural with the other catalysts in Table 8, we found that the studied catalysts obtained good results using only water as a solvent in 120 min of reaction. In addition, the time of reaction for 18%Nb-DB was half of those in the two reported studies [50,51]. Under slightly different reaction conditions, Vieira et al. [45] verified that the catalyst Nb_2_O_5_ converted 96.8% of xylose with 42.1% selectivity for furfural. When Nb_2_O_5_ was dispersed on silica (30% Nb_2_O_5_/SiO_2_), conversion decreased to 89.2%, with 37.2% selectivity for furfural. Thus, the quantity and strength of the moderate Lewis acid sites play a key role in transforming xylose into furfural.

## 4. Conclusions

The dealumination procedure followed by niobium loading improved the *BEA zeolite performance allowing the formation of new aluminum species, the creation of more Lewis acidity and the formation of secondary mesopores. This treatment succeeded in improving the zeolite performance in terms of the activity, selectivity, and stability in the catalytic dehydration of either ethanol or xylose. We can draw some essential and quantitative conclusions from Nb-DB catalysts. Ethanol dehydration was shown to be a good probe reaction due to the selectivity of products (ethylene and diethyl ether) depending on the catalyst structure and reaction conditions. Xylose dehydration could produce furfural, a biomass derivative that is a key molecule of chemical interest. Thermodynamic results also play a role of the separate pathways for the formation of the diethyl ether and furfural as predominant selective products. We found that 18%Nb-DB presented the secondary mesoporous and possessed high amounts of average strength acid sites, but a low amount of strong acid sites. A controlled dealumination process, followed by Nb impregnation, allowed the development of tailor-made Lewis and Brønsted acid catalysts, which provided pathways toward the selective formation of platform chemicals (diethyl ether and furfural) that were identified in the present manuscript. Under our experimental conditions, 18%Nb-DB catalyst was the most selective for diethyl ether production (97% at 61% conversion) and furfural (51% at 64% conversion). In addition, furfural was produced in water as solvent, which improved the green quality of any chemical process.

## Figures and Tables

**Table 1:** Nomenclature and description of the catalysts.

Catalyst	Description
HB	Protonic zeolite *BEA
DB	Zeolite *BEA dealuminated
10%Nb-DB	10% (wt.) Nb_2_O_5_ on zeolite *BEA dealuminated
18%Nb-DB	18% (wt.) Nb_2_O_5_ on zeolite *BEA dealuminated
25%Nb-DB	25% (wt.) Nb_2_O_5_ on zeolite *BEA dealuminated

**Table 2:** Main wavenumber (cm^−1^) bands identified in the FTIR of the catalysts.

Catalyst	A	B	C	D	E	F	G
HB	1221	1089	940	799	628	572	526
BD	1227	1097	960	802	623	575	525
10%Nb-DB	1224	1096	961	799	622	576	526
18%Nb-DB	1224	1096	958	799	623	576	526
25%Nb-DB	1227	1097	961	802	623	575	527

**Table 3:** Textural properties of the catalysts.

Catalyst	S_BET_^a^ (m^2^/g)	S_Ext_ (m^2^/g)	S_Micro_ (m^2^/g)	S_Meso_ (m^2^/g)	V_μ_ (cm^3^/g)	D_Meso_^a^ (nm)
HB	705	187	518	221	0.23	11.9
DB	533	153	380	201	0.17	14.5
10%Nb-DB	481	144	337	181	0.15	14.4
18%Nb-DB	443	156	287	178	0.13	12.3
25%Nb-DB	383	118	265	143	0.12	12.5

^a^ Standard deviations (2σ) S_BET_: ± 10 m^2^ g^−1^ and D_Meso_: ± 2 nm.

**Table 4:** Relative distribution of tetrahedral (Al-Td) and octahedral (Al-Oh) aluminum, and Q^4^ and Q^3^ environments of silicon, based on areas of Al signals and deconvolution of Si spectra.

Catalyst	Al-Td (%) ^a^	Al-Oh (%) ^a^	Si-Q^4^ (%) ^a^	Si-Q^3^ (%) ^a^
HB	64	36	77	23
DB	76	24	89	11
10%Nb-DB	66	34	77	23
18%Nb-DB	64	36	73	27
25%Nb-DB	57	43	81	19

^a^ The relative errors of the calculations are about 2% for Al and 3% for Si.

**Table 5:** The number of acid sites (n_i_, mmol g^−1^) and enthalpies (−ΔH_i_, kJ mol^−1^) of the sites obtained by microcalorimetry of Py adsorption, using a two-site model.

Catalyst	n_1_^a^	–ΔH_1_^b^	n_2_	–ΔH_2_
HB	0.21	105	0.41	62
DB	0.16	60	0.24	49
10%Nb-DB	0.21	71	0.18	51
18%Nb-DB	0.24	49	0.20	42
25%Nb-DB	0.23	42	0.17	40

^a^ Errors for n_i_ = ±0.02 mmol g^−1.^; ^b^ Errors for ΔH_i_ = ±2 kJ mol^−1^.

**Table 6:** Turnover number (TON) for ethanol conversion (TON_Conv._) (mole converted ethanol/mole acid sites), selectivity for diethyl ether (DEE) and TON_DEE_ (mole obtained DEE/mole acid sites) after 50 pulses of ethanol.

Sample	Temperature	Conv. (%) ^a^	TON_Conv._	Select. DEE (%) ^a^	TON_DEE_
HB	230	92	1.3	3	0.03
	250	93	1.3	4	0.05
	300	100	1.4	0	0
DB	230	71	1.5	66	1.00
	250	69	1.5	58	0.86
	300	81	1.7	35	0.61
10%Nb-DB	230	47	1.0	78	0.80
	250	55	1.2	63	0.76
	300	24	0.5	67	0.35
18%Nb-DB	230	61	1.2	97	1.15
	250	71	1.4	51	0.70
	300	84	1.6	13	0.21
25%Nb-DB	230	54	1.2	96	1.11
	250	45	1.0	73	0.70
	300	66	1.4	54	0.76

^a^ Data of conversion and selectivity for the samples HB, DB and 25%Nb-DB at 250 and 300 °C were reported in reference [9].

**Table 7:** Xylose conversion, TON_conv._ (mole converted xylose/mole acid sites), selectivity for furfural and TON_Furfural_ (mole obtained Furfural/mole acid sites) at temperatures of 160 °C and 180 °C.

Catalyst	Temp. (°C)	Conv.(%)	TON_Conv._	Select. Furfural (%)	TON_Furfural_
HB	160	43.5	23.4	37.1	8.7
	180	43.5	23.4	42.1	9.8
DB	160	25.0	20.8	60.2	12.5
	180	35.3	29.5	57.7	17.0
10%Nb-DB	160	22.1	18.9	48.5	9.2
	180	60.0	51.3	43.6	22.3
18%Nb-DB	160	47.8	36.2	38.0	13.8
	180	64.0	48.5	51.0	24.7
25%Nb-DB	160	65.0	54.1	38.5	20.8
	180	57.7	48.1	42.1	20.3

**Table 8:** Comparative conversion of xylose and selectivity for furfural using different catalysts and solvents, at specific temperatures and reaction conditions.

Catalyst	Reaction Conditions	Time (min)	Temperature (°C)	Conversion (%)	Selectivity (%)	Ref.
Cu/SBA-15-SO_3_H	H_2_O-n-butanol (1/3 v ratio) and 3MPa H_2_	360	130	93.7	10.7	[50]
H-BEA	H_2_O	240	140	51.5	36.9	[51]
H-BEA	DMSO	240	140	89.7	26.7	[51]
H-ZSM-5	H_2_O	240	140	56.8	30.2	[51]
H-ZSM-5	H_2_O	240	140	69.3	29.6	[51]
Nb_2_O_5_	THF/H_2_O 4/1	120	130	95.4	46.8	[45]
Nb_2_O_5_	H_2_O	120	130	96.8	42.1	[45]
DB	H_2_O	120	160	25	60	This work
18%Nb-DB	H_2_O	120	180	64	51	This work

## Supplementary Materials

The following are available online at https://www.mdpi.com/2079-4991/10/7/1269/s1. Figure S1: HPLC-RID chromatograms of xylose and furfural standards, Figure S2: N_2_ isotherms of adsorption/desorption (−196 °C) of the catalysts, Figure S3: Deconvolution of the 29Si MAS NMR spectra, Figure S4: FTIR spectra (1500 to 1400 cm^−1^) of pyridine adsorbed on the catalysts, Figure S5: Conversion (%) of ethanol using a pulsed-flow fixed-bed microreactor coupled to a gas chromatograph system (pulses 1 and 50), Figure S6: Selectivity for diethyl ether (DEE) and ethylene using a pulsed-flow fixed-bed microreactor coupled to a gas chromatograph system (pulses 1 and 50).
