# Supplementary material for: Niobium on BEA Dealuminated Zeolite for High Selectivity Dehydration Reactions of Ethanol and Xylose into Diethyl Ether and Furfural
Source: Nanomaterials (Basel). 2020 Jun 29;10(7):1269. doi: 10.3390/nano10071269 (PMC7407447; doi:10.3390/nano10071269)
Supplement: Supplementary file 1 [file nanomaterials-10-01269-s001.pdf]

Supplementary Material

Article: Niobium on BEA dealuminated zeolite for high selectivity dehydration reactions of ethanol and xylose into diethyl ether and furfural

Authors: Deborah S. Valadares, Maria C. H. Clemente, Elon F. Freitas, Gesley A. V. Martins, José A. Dias, Silvia C. L. Dias*

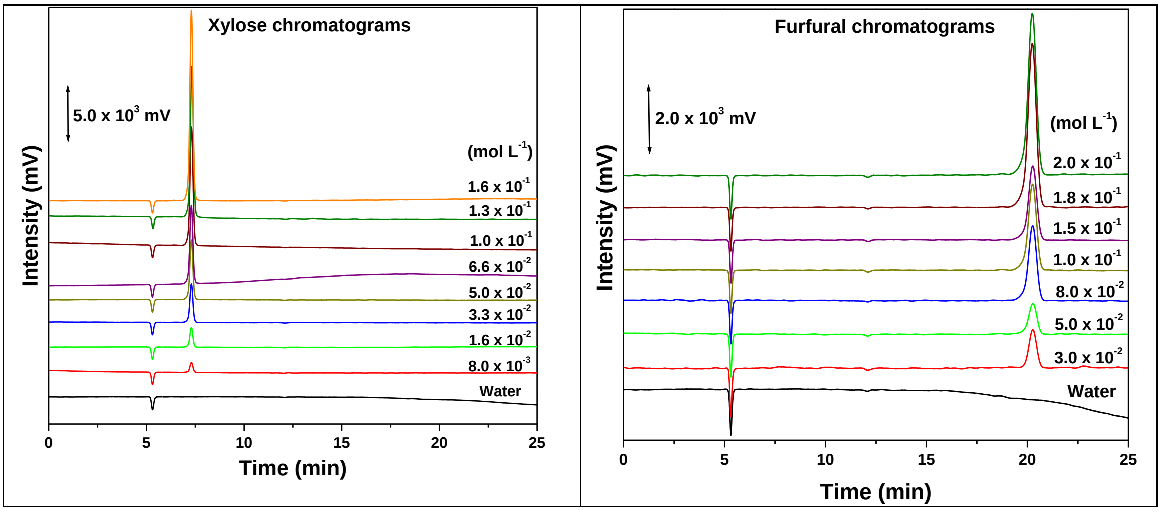

Fig. S1. HPLC-RID chromatograms of xylose and furfural standards.

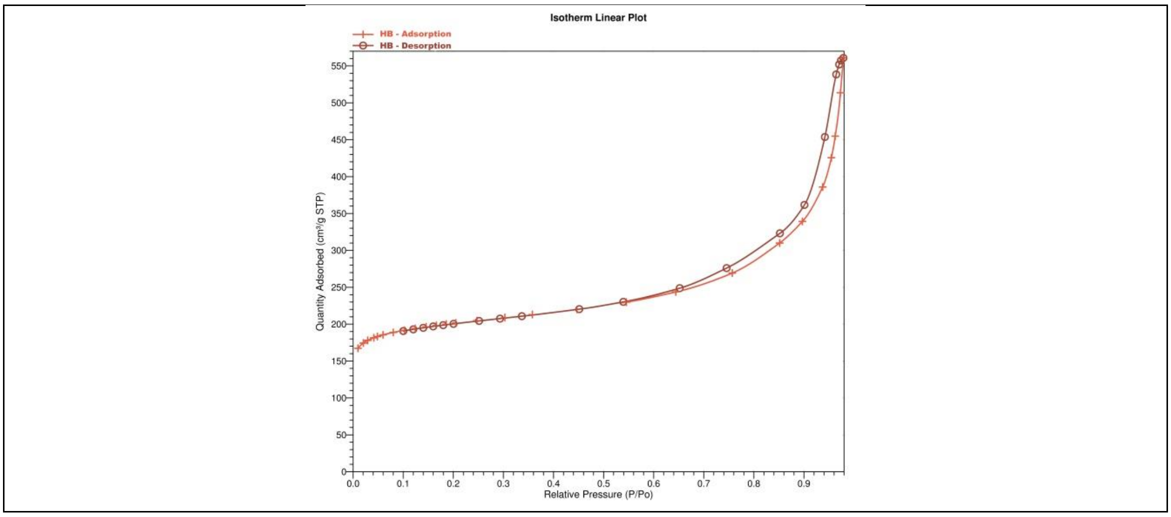

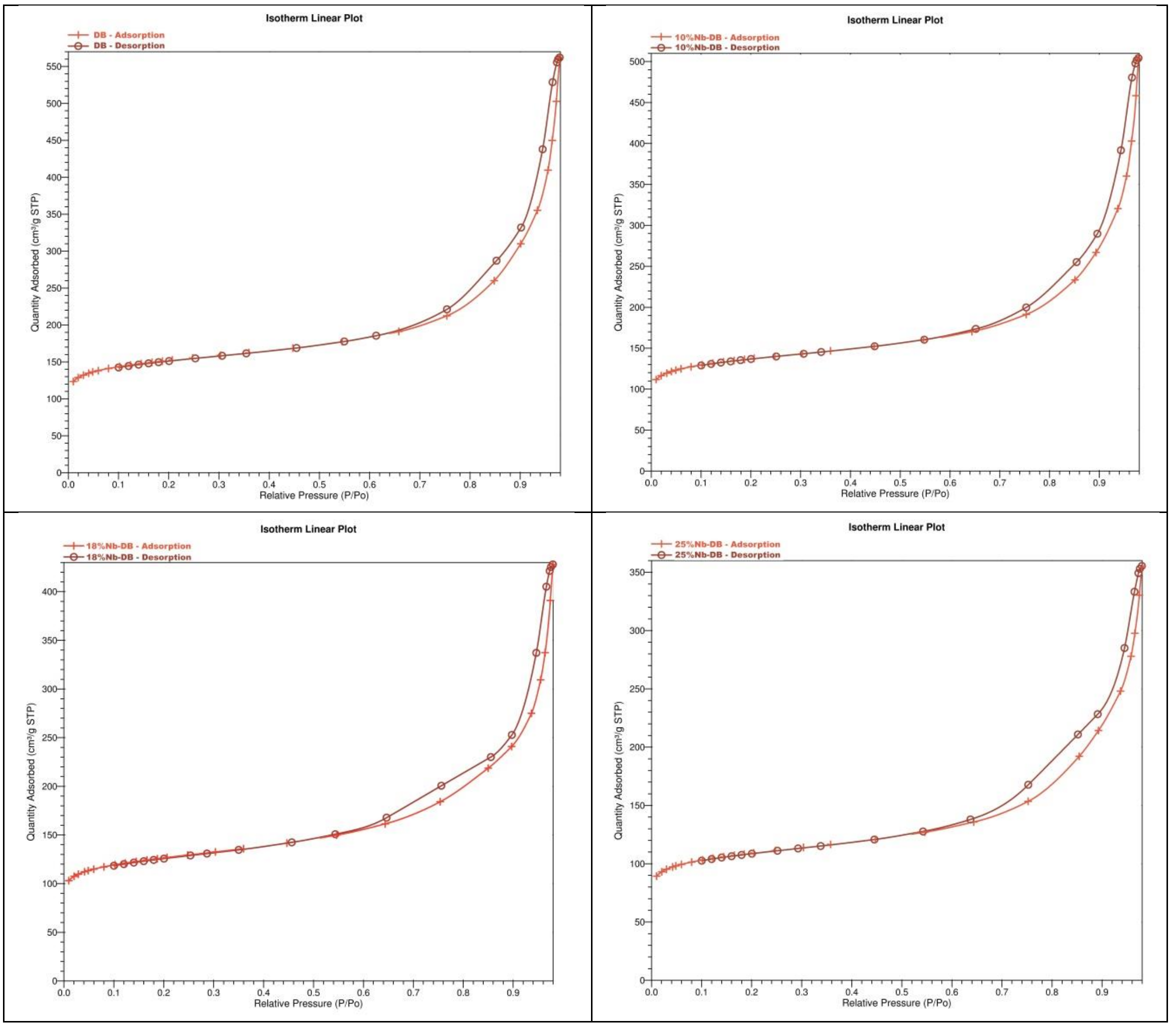

Fig. S2. N_2 isotherms of adsorption/desorption (-196 °C) of the catalysts.

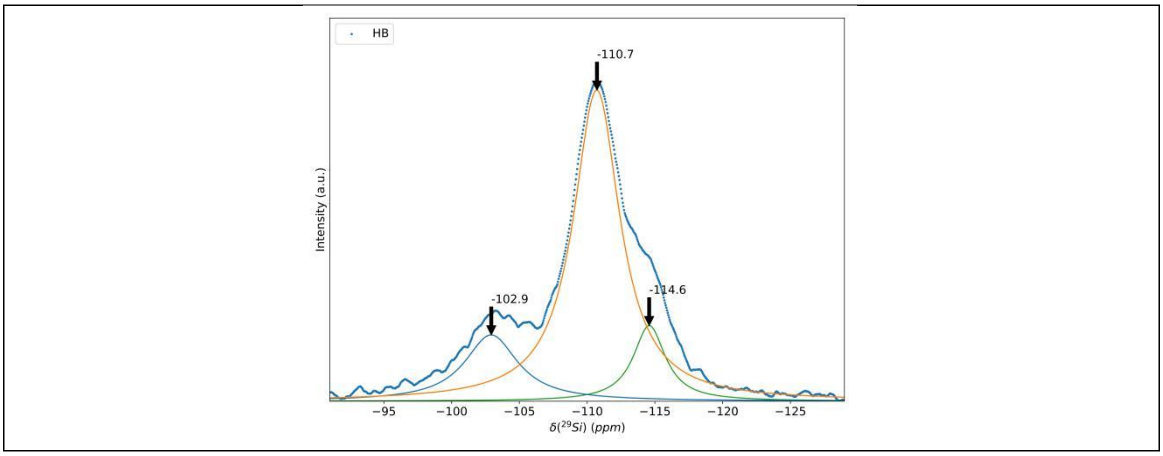

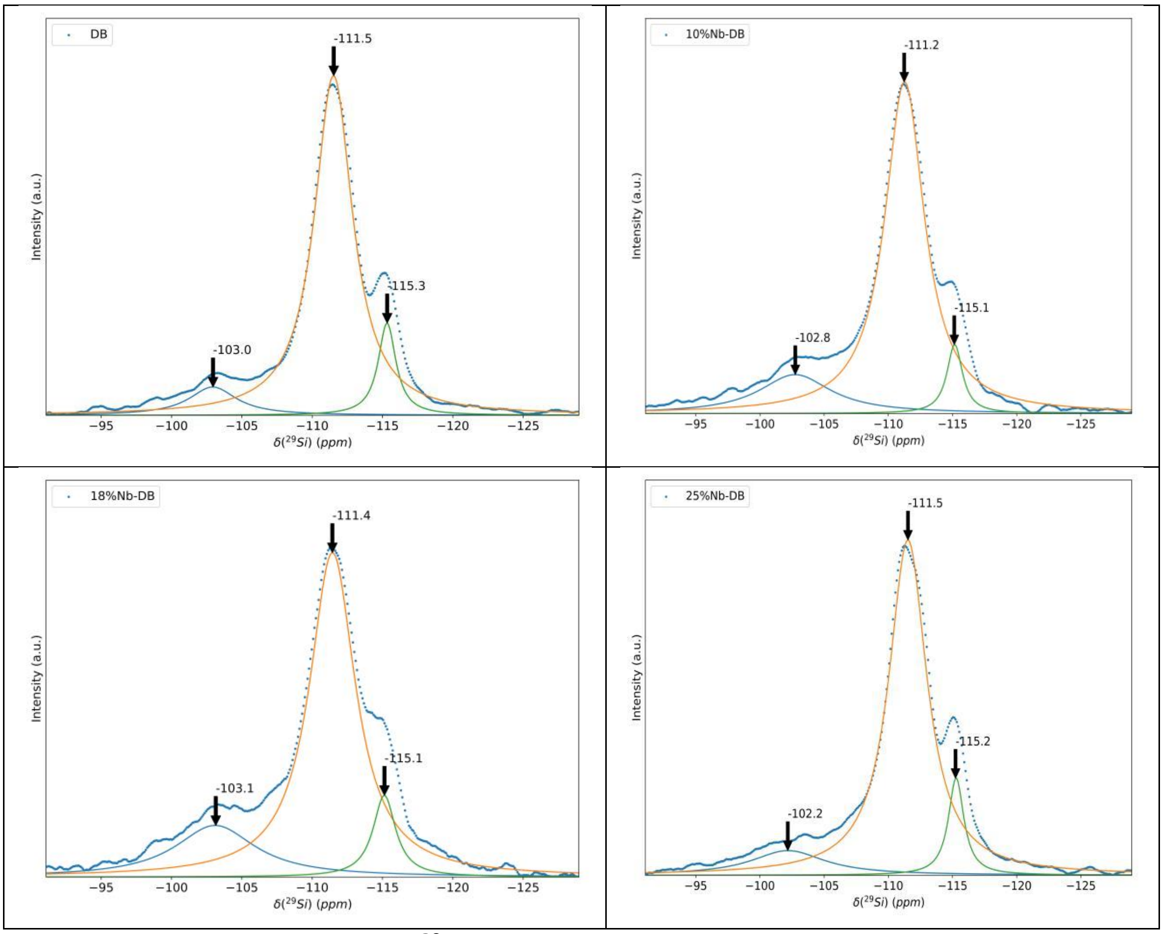

Fig. S3. Deconvolution of the ^{29}Si MAS NMR spectra. The following conditions were used: LB = 10 Hz; smoothing with 25 pt.; three signals using Lorentzian fittings (autofitting); Software Python (v. 3.7 with the Scipy library v. 1.4.1).

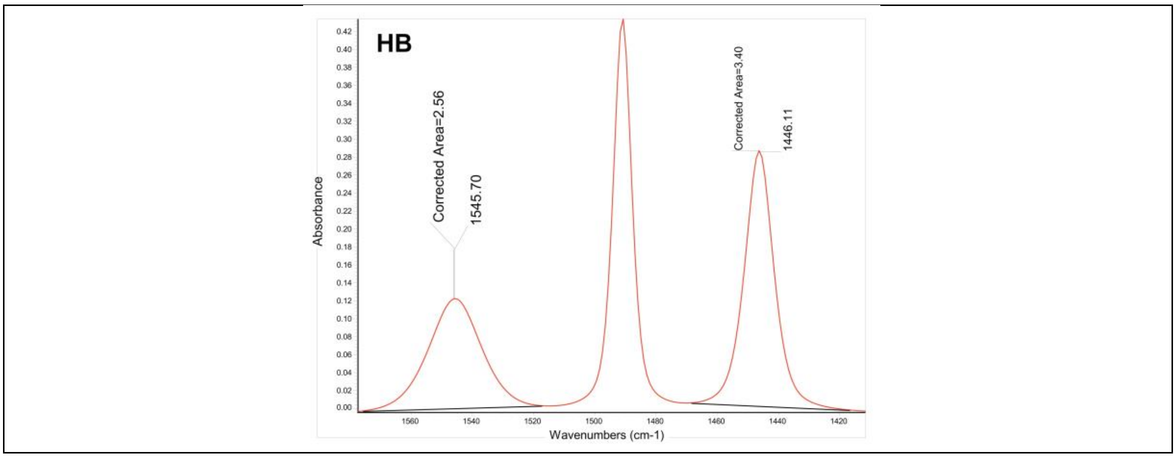

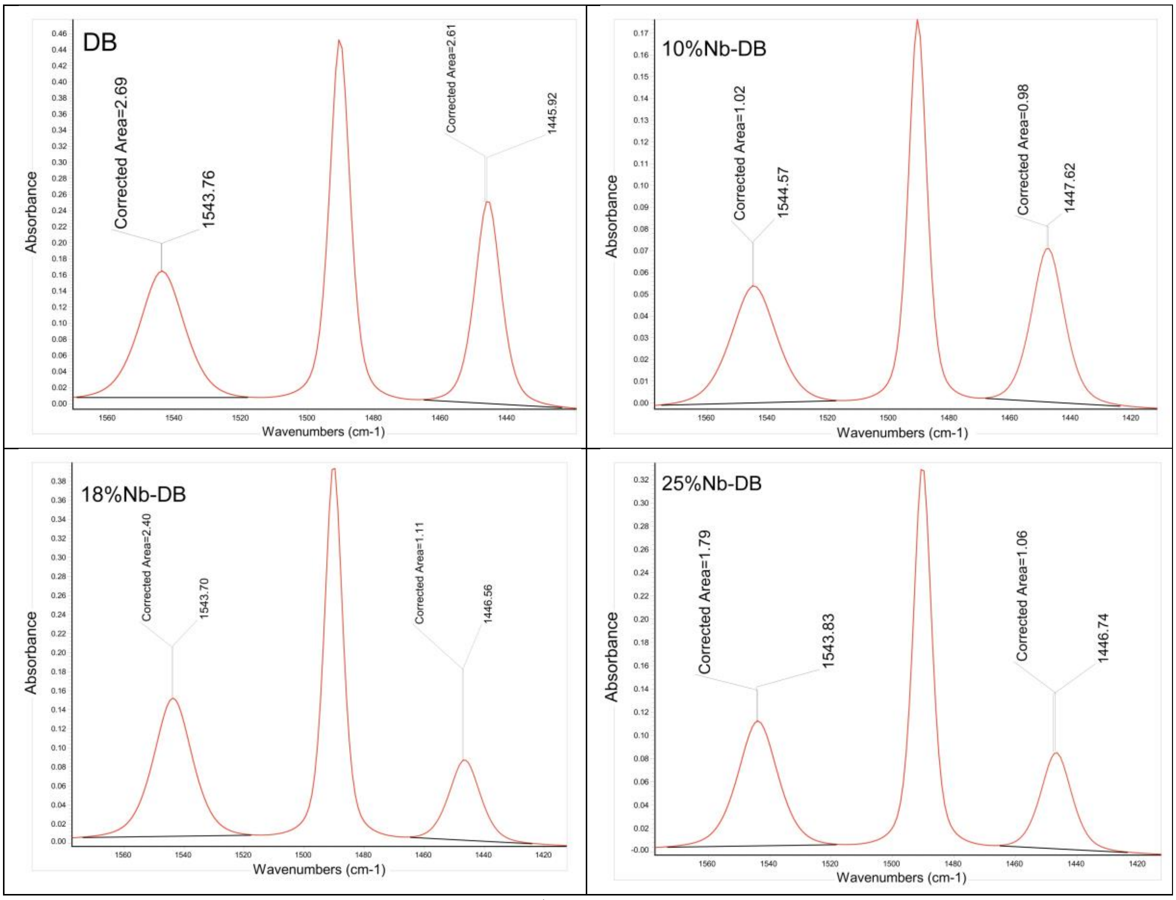

Fig. S4. FTIR spectra (1500 to 1400 cm^{-1}) of pyridine adsorbed on the catalysts.

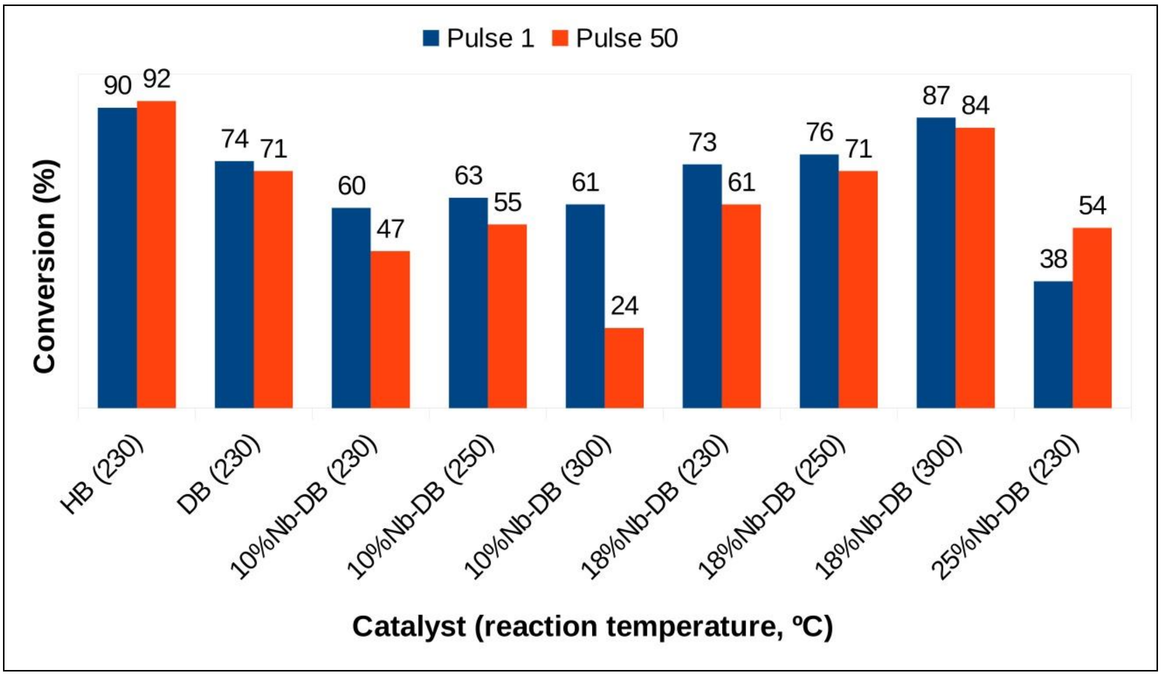

Fig. S5. Conversion (%) of ethanol using a pulsed-flow fixed-bed microreactor coupled to a gas chromatograph system (pulses 1 and 50).

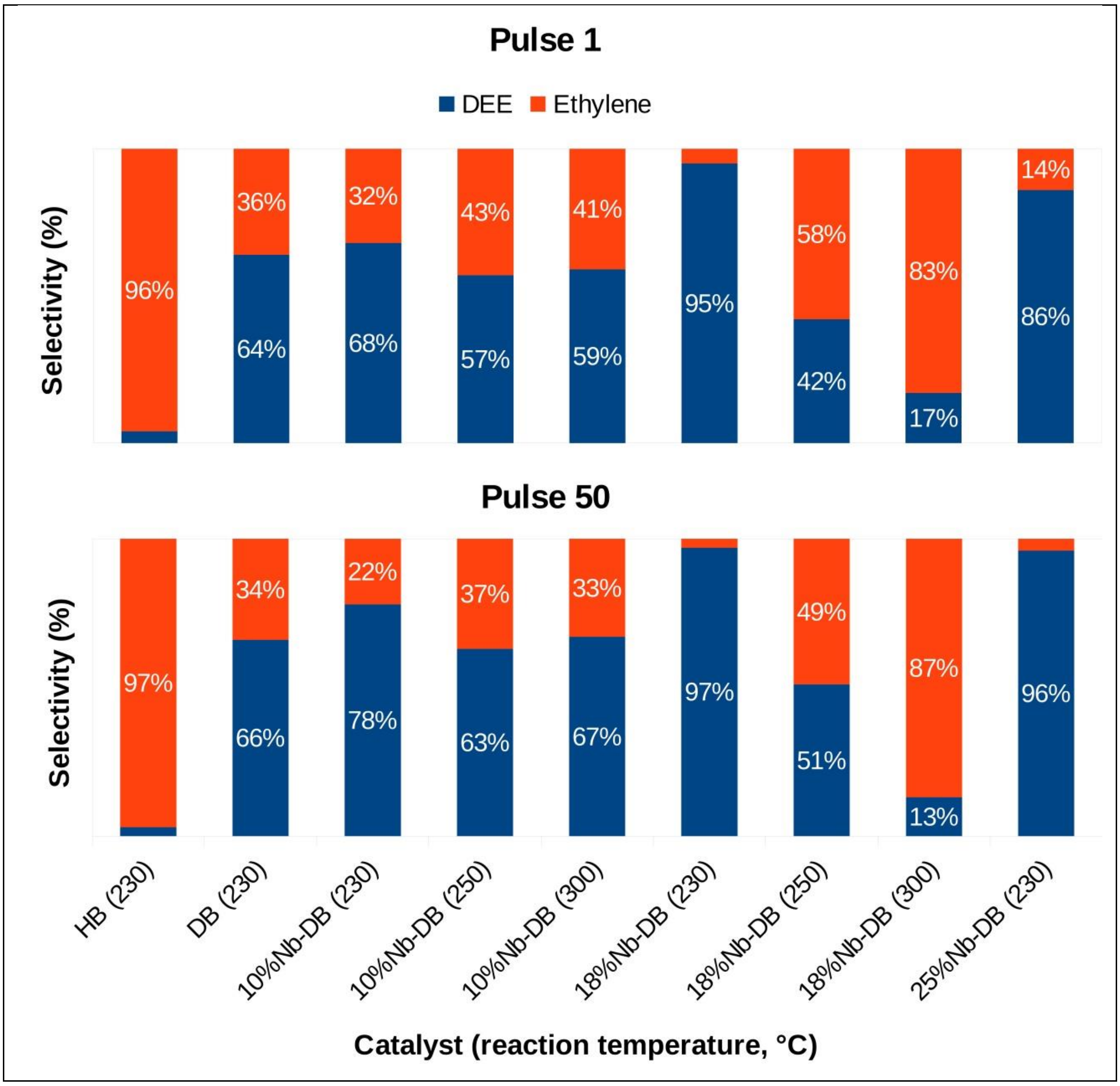

Fig. S6. Selectivity for diethyl ether (DEE) and ethylene using a pulsed-flow fixed-bed microreactor coupled to a gas chromatograph system (pulses 1 and 50).
